# Patient-reported geriatric syndromes and their association with quality of life: findings from a cross-sectional study in German older adults

**DOI:** 10.1007/s41999-025-01332-7

**Published:** 2025-10-16

**Authors:** Aline Schönenberg, Konstantin G. Heimrich, Annika Sternkopf, Paul Lochbihler, Tino Prell

**Affiliations:** 1 Department of Geriatrics, Halle University Hospital, Halle (Saale), Germany; 2 Department of Geriatrics, Jena University Hospital, Jena, Germany

**Keywords:** Geriatric syndromes, Multimorbidity, Patient-centered, Older adults, Geriatrics, Geriatric assessment

## Abstract

**Purpose:**

Many older patients suffer from multimorbidity, rendering disease-specific approaches moot. Instead, overarching geriatric syndromes can be used to describe patients’ experiences above and beyond concrete diagnoses. However, little is known about how patients themselves perceive these geriatric syndromes.

**Methods:**

We collected self-reported data on *N* = 511 (83.0 ± 5.96 years, 66% female) geriatric in- and *N* = 155 outpatients (79.0 ± 6.42 years, 60% female) on the occurrence of nine geriatric syndromes: reduced mobility, falls, problems with cognition, depressiveness, loneliness, pain, incontinence, problems with sleep, and problems with swallowing. We additionally asked about the perceived restriction and expectations regarding improvement of the geriatric syndromes. Using descriptive statistics, group comparisons, and linear regression, we describe how patients perceive and experience geriatric syndromes and their association with mental and physical quality of life (QoL) while controlling for cognition, functional status, and health.

**Results:**

On average, patients report 3.4 (SD = 1.8) of nine different geriatric syndromes, while 47.2% reported ≥ 4. The most frequent geriatric syndromes were mobility problems, falls, and pain; these were also perceived as most restrictive in daily life. A higher number of geriatric syndromes significantly reduces mental and physical QoL, above and beyond physical health. For physical QoL, mobility problems, falls, and pain are most influential, while mental QoL is linked with depressiveness, loneliness, and sleep problems. These associations were even stronger in outpatients than in inpatients.

**Conclusion:**

Geriatric syndromes are highly prevalent and lead to reduced mental and physical QoL. As they impact QoL above and beyond physical health and functionality, geriatric syndromes and their association with age-related expectations should be incorporated in clinical care to improve well-being.

**Supplementary Information:**

The online version contains supplementary material available at 10.1007/s41999-025-01332-7.

## Introduction

Geriatric medicine has undergone a notable shift in its approach, away from the management of individual diseases towards a comprehensive focus on overall geriatric syndromes [1–3]. This shift is driven by the high prevalence of multimorbidity, wherein patients frequently present with multiple health concerns [2, 4–6]. Geriatric syndromes can be interpreted as cumulative effects of multiple impairments, leading to increased overall vulnerability of older adults [1]. Consequently, healthcare providers are confronted with the challenge of managing the intricacies of these geriatric syndromes, which encompass a range of issues including mobility difficulties, cognitive decline, incontinence, depressive mood, polypharmacy, malnutrition, sleep disorders, and others that collectively impact the quality of life (QoL) of older patients [5–8]. Of note, no concrete definition of geriatric syndromes exists due to their highly heterogeneous and multifaceted nature [9, 10]. These heterogeneous definitions leave the selection of geriatric syndromes open to the respective researchers. Despite this heterogeneity in the included geriatric syndromes, their impact on well-being and QoL is unmistakable across studies [11, 12]. The presence of geriatric syndromes may lead to hospitalization or institutionalization [2, 8, 13] and may even increase mortality rates [14, 15]. In patients with diabetes, geriatric syndromes have a negative impact on both physical and mental QoL [16]. In patients with chronic kidney diseases, this association between QoL and geriatric syndromes even remained present when taking concrete illnesses into account [17]. This influence on QoL is detrimental, as QoL and subjective well-being become a focal outcome in geriatric care, where full recovery is not feasible [18–20].

Despite the clinical importance of geriatric syndromes, research on their prevalence and impact is rare, potentially due to the challenges of scientific recruitment in geriatric patients and the overall underrepresentation of geriatric patients in medical studies [21]. In addition, the majority of studies on geriatric syndromes is informed by external information taken from medical assessments, diagnoses, or objective measurements [6, 15, 22], but the patient perspective has not yet been considered. As expectations and experiences shape patients’ health behavior and health outcomes [23], it is crucial to also assess geriatric syndromes as patient-experienced symptom clusters. While geriatric syndromes may pose a challenge for healthcare providers, as they do not allow for a differentiation between the overlapping causes of the geriatric syndromes, they may aid older patients in describing their actual daily challenges. For example, a patient may suffer from Parkinson’s disease, polyneuropathy, osteoporosis, and a fracture after a fall; while it is important for medical staff to understand the causes of each geriatric syndrome, the primary issue experienced by this patient is a restriction in mobility, irrespective of the underlying illnesses. Thus, it is imperative to comprehend the patient perspective to develop patient-centered care strategies that integrate both the clinical aspects of geriatric syndromes and the patients’ own experiences. This is especially important as the prevalence of geriatric syndromes rises with advancing age [2, 5, 24], cumulating in as many as 90% of geriatric patients reporting any geriatric syndromes, while most report multiple [6, 7, 22].

This manuscript, therefore, aims to explore how geriatric patients experience selected geriatric syndromes, including their perceptions of the presence, limitations in daily life, and potential for improvement. For this purpose, we asked geriatric patients about their perception of nine geriatric syndromes, including mobility problems, falls, cognitive problems, depressiveness, loneliness, pain, incontinence, sleep disturbances, and dysphagia. Through this lens, we seek to provide insights into improved delivery of geriatric care by incorporating the patients’ voices into the management of their health.

## Methods

### Patients and recruitment

Data was collected as part of the *SelfManGer—Self-management of geriatric patients in Germany* study [25] conducted at geriatric wards in two hospitals (trial registration: DRKS00031016) in Saxony-Anhalt and the related *JenaGer* Study conducted in Thuringia. While having differing overall aims, the *SelfManGer* and *JenaGer* studies were designed in concordance with overlapping instruments to ensure two comparable core datasets. In addition, for *SelfManGer*, patients were recruited from two collaborating GP practices in urban areas.

Data collection took place between February 2023 and August 2024. We included (1) older inpatients receiving comprehensive geriatric care within the German Operation and Procedure Classification System (OPS 8–550) and (2) older outpatients (65 years and older with multimorbidity). The exclusion criteria were severe dementia or acute delirium, severe depression, and being fully dependent in activities of daily living according to the Barthel Index [26]. Except for diagnosed dementia and acute delirium, we did not specify a cut-off for cognitive tests as an exclusion criterion but instead included patients if study staff felt they were able to hold a meaningful conversation and comprehend the questionnaires [27].

All patients were screened based on their medical records, and then trained study staff approached eligible patients and informed them about the study. The study was approved by the local ethics committee of Halle University Hospital, the State Chamber of Physicians of Saxony-Anhalt (Landesärztekammer; approval number: 2022–026), and the ethics committee of Jena University Hospital (2023–2923-BO). If the patients gave their written informed consent, study staff proceeded to go through the questionnaires together [27]. The study included both questionnaires and medical information obtained from the geriatric assessments *performed* as part of routine inpatient care.

The following questions were covered in a questionnaire; for transparency and traceability, an English translation is available along with the data [28]

- Sociodemographic information: age, gender, living situation (alone–not alone), martial state (married or in partnership—single—widowed or divorced), education (low: less than 8 years, middle = 8 to 10 years, high = more than 10 years);
- Geriatric syndromes:
  o Presence of (yes/no): impaired *mobility*, *falls*, problems with *cognition,* sadness/*depressiveness*, *loneliness*, *pain*, *incontinence*, problems with *sleep*, problems with swallowing (*dysphagia)*
  o Which of the above mentioned syndromes is the most restrictive to you in your daily life? (single choice)
  o On a scale of 0 to 100, how restricted are you in your daily life because of this problem? (100 indicates no restriction at all, 0 indicates fully restricted)
  o On a scale of 0 = *sure that it will not improve* to 100 = *sure that it will definitely improve*, how confident are you that the problem will improve?
- *QoL* according to the WHOQOL-Bref questionnaire [29, 30]; in this analysis we focus on the physical and mental QoL subscales.

The geriatric syndromes were selected by an expert panel of geriatric specialists and a multidisciplinary team (psychologist, occupational therapists, and nurses) based on syndromes addressed in previous research [5, 8, 22]. Previously assessed syndromes were chosen based on patient relevance. Since this study takes a patient-centered approach, the aim was to select syndromes that were considered relevant and palpable for patients, excluding syndromes such as multimorbidity, malnutrition, or polypharmacy. These syndromes primarily reflect healthcare professionals’ assessments and can be measured objectively. We also excluded overall frailty for the patient assessment as this concept was believed to be too complex for the purpose of this study. Likewise, the expert team set out to include both physical and mental geriatric syndromes such as depressive mood, loneliness, and cognitive decline, to cover many of the known factors influencing well-being in advancing age [31–33].

In addition to this questionnaire, the following variables were extracted from each inpatient’s medical record:

- Geriatric depression scale, *GDS* [34, 35], ranging between 0 and 15 points with higher scores indicating higher levels of depressiveness
- *Barthel* Index [26, 36], ranging between 0 and 100 points with higher scores indicating independence in daily activities
- Geriatric screening according to *Lachs *[37, 38] depicting 15 areas of potential restriction in daily tasks such as movement, cognition, incontinence or mood, with higher scores indicating a higher level of pathology
- Cognition according to the Montreal Cognitive Assessment, *MoCA* or the Mini Mental Status Test *MMST* [39–42]. Of note, for reasons of comparability, the MoCA was transformed to match the MMST according to the conversion described in Fasnacht et al. [43]. For both assessments, higher scores indicate better cognitive performance
- Number of different medications taken daily, *Nmeds*

For the outpatients recruited from GP practices, no routine geriatric assessment was available. To describe their mental and cognitive characteristics, we performed the *GDS* and the *MiniCog* [44]. The MiniCog combines word recall and clock test. A score below three indicates cognitive impairment.

In total, *N* = 666 patients were recruited and completed the questionnaire of interest on geriatric syndromes, *N* = 511 of which were inpatients and *N* = 155 outpatients recruited from the GP practices.

### Statistical analysis

We described the included patients grouped by location (inpatient vs outpatient) with descriptive statistics using mean (M) and standard deviation (SD) as well as median (MD) and interquartile range (IQR) in case of non-normal distribution as assessed by the Shapiro–Wilk test. For group comparisons regarding gender and geriatric syndromes, we used the Chi^2^ test for categorical variables as well as the Mann–Whitney U test or Kruskal–Wallis test with Dunn test and Bonferroni correction. Effect sizes for metric data were calculated as rank biserial correlation *r* and for Chi^2^ test as Cohen’s ω. Values for both can be considered low between |0.1| and <|0.3|, medium between |0.3| and <|0.5|, and high when >|0.5| [45, 46].

Initially, we describe the presence of the respective geriatric syndrome as well as their co-occurrence, the amount of limitation patients experience due to the respective geriatric syndrome, and expectations regarding improvement.

Finally, we assessed the impact of geriatric syndromes on physical and mental QoL using the WHOQOL-Bref questionnaire. Using regression analysis, we first defined the Number of Syndromes as a single independent variable in a simple model, followed by the inclusion of age and gender, and then we determined the robustness of the association with the addition of the objective health-related covariates GDS, MMST/MiniCog, and for inpatients Lachs. Using group comparison, we analyzed differences in patients’ QoL depending on the presence of each geriatric syndrome.

All analyses were performed in R Version 4.3.0 with a significance value of 0.05 and a two-sided approach. Missing data in covariates were treated according to list-wise deletion, meaning that only complete observations were used for each analysis.

## Results

### Descriptive statistics

The characteristics of the cohort are given in Table 1**.** We included *N* = 511 geriatric inpatients and *N* = 155 geriatric outpatients. The inpatients were 83 years old (SD = 5.96), ranging from 67 to 96 years, while the outpatients were younger at 79 years (SD = 6.42). Most patients were female, lived alone as widows, and had at least 10 years of education. With a GDS score of 4.09, the patients did not show signs of a depressive disorder, and an average of 25.20 points in the MMST (range = 10–30) indicated mild cognitive deficits.

**Table 1:** Descriptive statistics of the included patients

Variable	Inpatient	Outpatient	Comparison^a^
	M (SD)	M (SD)	*p*, r
Age	83.0 (5.96)	79.0 (6.42)	< 0.001, 0.35
Number of Syndromes	3.61 (1.79)	2.74 (1.85)	< 0.001, 0.29
Number of Medications	10.4 (4.72)	7.8 (4.12)	< 0.001, 0.32
MMST/MiniCog^b^	25.2 (3.31)	3.16 (1.56)	
GDS	4.09 (3.00)	4.4 (3.46)	0.454, -0.04
Barthel Index (admission)	46.3 (18.8)		
Lachs	6.72 (2.12)		
WHO_physical	53.1 (21.5)	61.5 (20.1)	< 0.001, -0.26
WHO_mental	68.3 (16.7)	67.0 (16.9)	0.596, 0.03
	Count (%)	Count (%)	*p*, W
Gender: Female	335 (65.56)	92 (60.0)	242, 0.049
0	195 (38.69)	99 (67.81)	
1	50 (9.92)	16 (10.96)	
2	154 (30.56)	19 (13.01)	
3	92 (18.25)	12 (8.22)	
4	11 (2.18)	0	
5	2 (0.40)	0	
Health Satisfaction			< 0.001. 0.218
1 very dissatisfied	68 (14.35)	5 (3.25)	
2 dissatisfied	165 (34.81)	52 (33.77)	
3 neither	80 (16.88)	27 (17.53)	
4 satisfied	137 (28.90)	70 (45.45)	
5 very satisfied	24 (5.06)	0	
Number of Medications > 5	413 (85.51)	101 (66.45)	< 0.001, 0.207

MMST, Mini mental status test (cognition); GDS, geriatric depression scale; WHO_, WHOQOL-Bref quality of life questionnaire; mental and physical scale; Health Satisfaction, WHOQOL-Bref Item 2 (“How satisfied are you with your health?”)

^a^Group Comparison between in- and outpatients

^b^MMST Range 0–30, with higher values indicating better cognitive performance. For outpatients MMST was not available, the MiniCog was performed as a brief screening containing clock test and recall. Scores ≤ 2 indicate risk for cognitive impairment (range 0–5)

Effect size: Wilcoxon Rank Sum/Mann Whitney Test for metric data: r = rank biserial correlation, Chi2 Test: Cohen’s ω

### Occurrence of geriatric syndromes

**Fig. 1:**
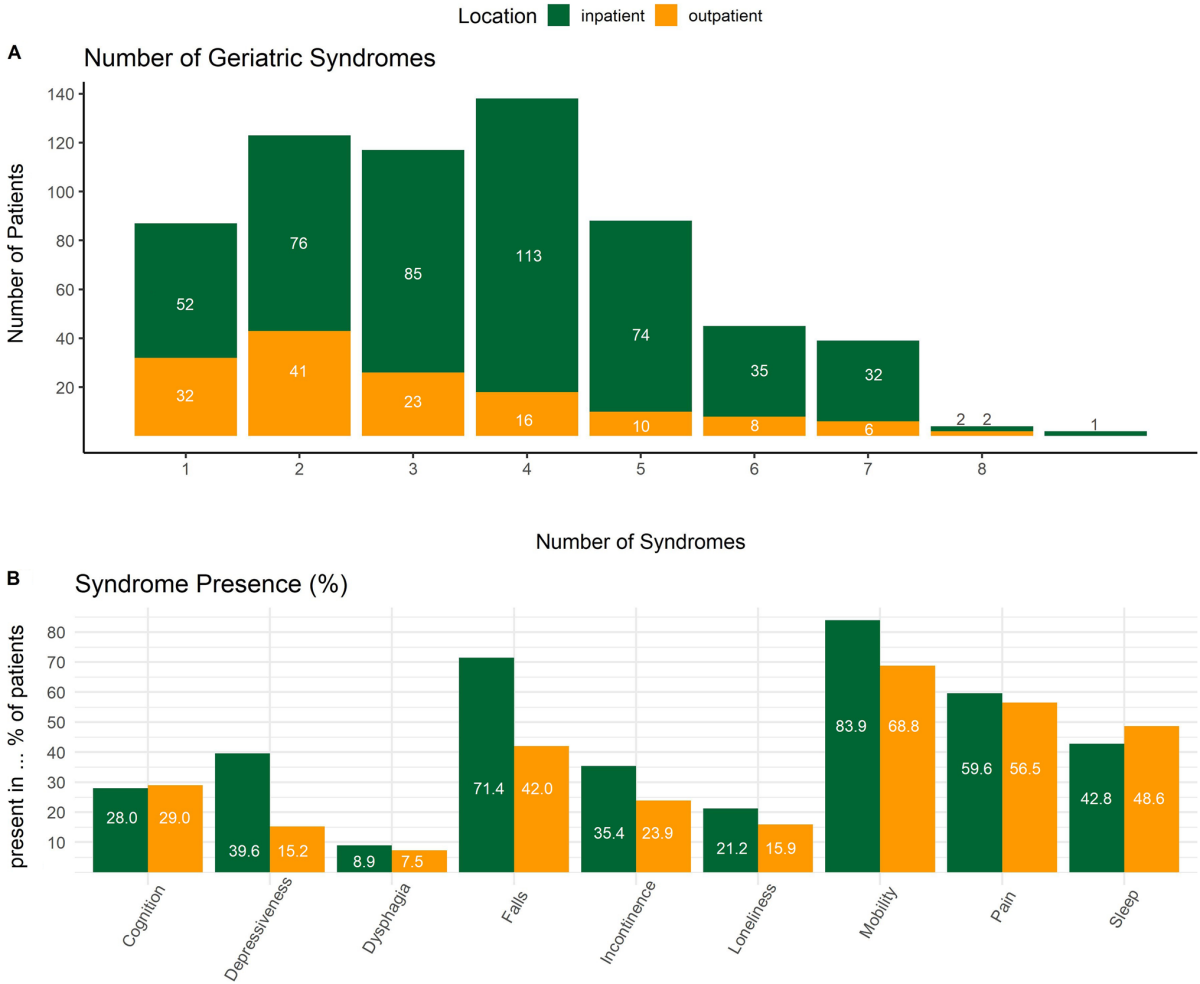
Absolute syndrome number and presence for in- and outpatients

On average, patients reported to have 3.41 (SD = 1.84) of the nine geriatric syndromes. Inpatients had more geriatric syndromes (M = 3.61) compared to outpatients (M = 2.74) (Table 1**)**. 53.23% of inpatients reported 4 or more geriatric syndromes. Only 10% of inpatients and 21% of outpatients reported a singular geriatric syndrome (Fig. 1A). The most common geriatric syndromes were problems with mobility, pain, falls, and sleep (Fig. 1B, Supplement Table 1).

Regarding gender, we found significant differences in the occurrence of geriatric syndromes only for pain (*p* = 0.009), with 57.94% of females reporting pain compared to 47.06% of males; dysphagia (*p* = 0.017) was more commonly reported by men (11.34%) than women (5.84%). A similar pattern remained after splitting by recruitment location (Supplement Table 2), with female inpatients (58.5%) reporting more pain than male (48.9%) inpatients (*p* = 0.047).

**Table 2:** Regression analysis on association between Number of Syndromes and Quality of Life

Predictors	Est	CI	P	Predictors	Est	CI	ß
Inpatients–Physical QOL				Outpatients–Physical QOL			
(Intercept)	85.41	56.23 – 114.6	** < 0.001**	(Intercept)	75.91	44.77 – 107.05	** < 0.001**
SyndromNum	-4.15	-5.20 – -3.10	** < 0.001**	SyndromNum	-5.49	-7.36 – -3.63	** < 0.001**
age	0.24	-0.05 – 0.53	0.106	age	0.03	-0.35 – 0.42	0.862
gender [male]	0.29	-3.27 – 3.85	0.872	gender [male]	1.56	-3.44 – 6.56	0.537
GDS	-1.23	-1.84 – -0.62	** < 0.001**	GDS	-2.07	-3.10 – -1.03	** < 0.001**
MMST	-0.84	-1.35 – -0.33	**0.001**	MiniCog	1.26	-0.41 – 2.94	0.137
LACHS	-1.63	-2.47 – -0.79	** < 0.001**				
*N* = 474, R^2^ / R^2^ adjusted0.269 / 0.260				*N* = 124, R^2^ / R^2^ adjusted0.567 / 0.549			
Inpatients–Mental QOL				Outpatients–Mental QOL			
(Intercept)	64.85	42.03 – 87.67	** < 0.001**	(Intercept)	75.38	54.62 – 96.14	** < 0.001**
SyndromNum	-2.72	-3.53 – -1.91	** < 0.001**	SyndromNum	-1.71	-2.87 – -0.56	**0.004**
age	0.25	0.02 – 0.48	**0.030**	age	0.11	-0.15 – 0.36	0.414
gender [male]	-0.21	-3.00 – 2.57	0.880	gender [male]	1.34	-1.98 – 4.65	0.427
GDS	-1.80	-2.28 – -1.32	** < 0.001**	GDS	-3.15	-3.79 – -2.52	** < 0.001**
MMST	0.10	-0.31 – 0.51	0.634	Minicog	0.30	-0.78 – 1.38	0.581
LACHS	-0.40	-1.06 – 0.25	0.230				
*N* = 472, R^2^ / R^2^ adjusted0.278 / 0.269				*N* = 139, R^2^ / R^2^ adjusted0.682 / 0.670			

Significant values of p < .05 are denoted in bold. GDS, Geriatric Depression Scale; MMST, Mini Mental Status Test; Minicog, Mini Cognitive Assessment; Lachs, Geriatric Screening according to Lachs; SyndromNum, Number of geriatric syndromes

### Perceived restrictions because of geriatric syndromes

**Fig. 2:**
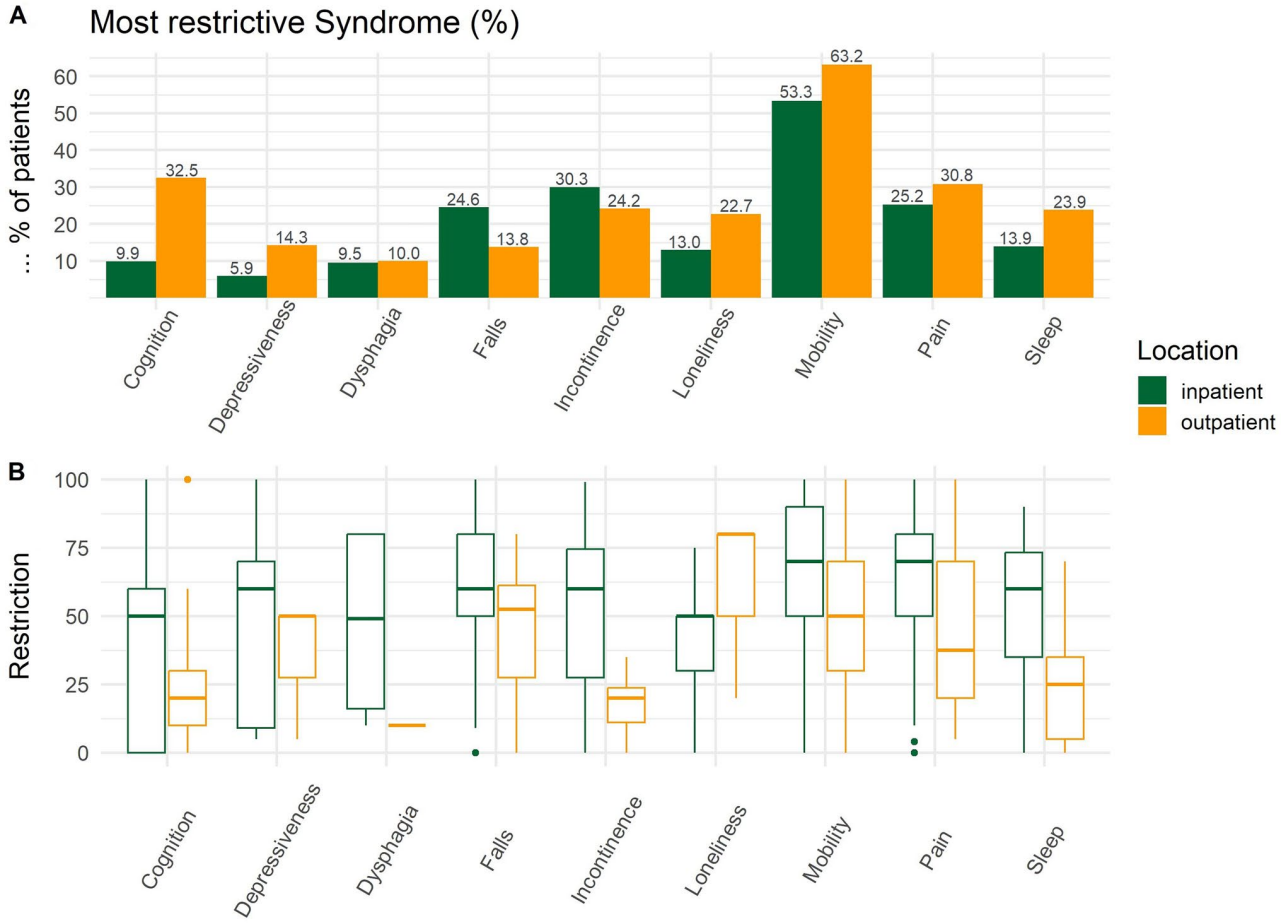
Syndrome restrictions

Of all the geriatric syndromes, 271 (44.7%) patients selected mobility problems as the most restrictive geriatric syndromes in their daily lives, followed by pain (95, 15.7%) and falls (91, 15.0%). When looking at in- and outpatient separately, 53.2% of inpatients with mobility problems selected those as most impactful; this association was even stronger in outpatients, where 63.2% rated their mobility problems as most restrictive (Supplement Table 1). Almost a third of outpatients rated their cognitive impairments as most restrictive, while only 10.0% of inpatients did so (Fig. 2A), despite a higher mean restriction in inpatients (M = 37.3) than outpatients (M = 24.9). In contrast, inpatients rated incontinence (29.3%) and dysphagia (38.2%) as more restrictive than outpatients did (12.6% and 22.5%, respectively). The amount of perceived restriction was lower in outpatients than inpatients for most geriatric syndromes except loneliness (Supplement Table S1, Fig. 2B).

As shown in Fig. 2B and Supplement Table S1, there was considerable heterogeneity in the restrictions patients experienced due to the geriatric syndrome, especially in outpatients. Most patients who selected mobility and pain-related geriatric syndromes experienced those as rather restrictive, while problems with cognition, swallowing, and sleep were considered relatively less restrictive.

### Perceptions of potential for improvement

**Fig. 3:**
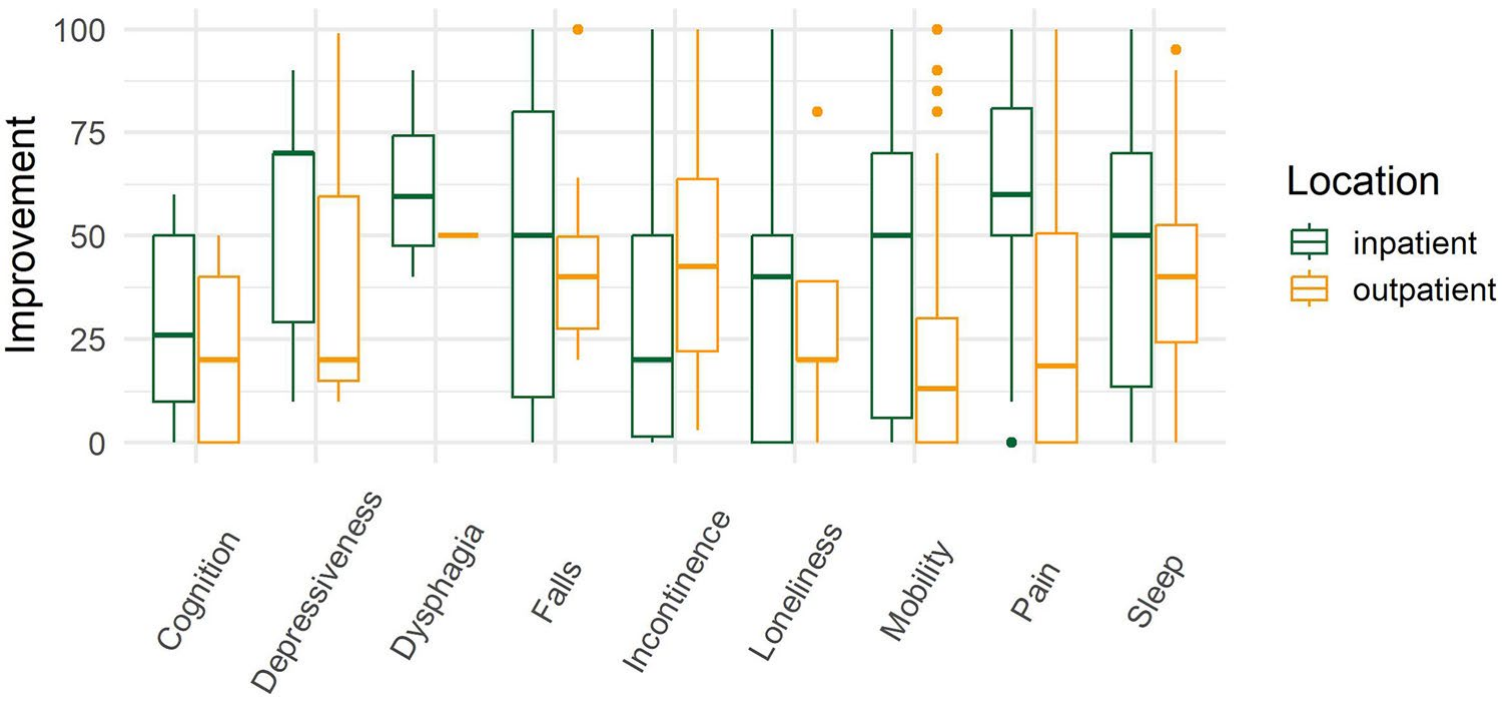
Expectations regarding the improvement of syndromes

In addition to inquiring about the restriction, patients were asked to indicate whether they expected the respective geriatric syndrome to improve (*0* = *sure it will not improve*, *100* = *sure it will definitely improve*). Substantial heterogeneity in the patients’ responses was observed when utilizing a cutoff of 50 (50 = undecided, < 50, > 50). For mobility problems as the most prevalent geriatric syndrome, 41.6% of inpatients but only 15% of outpatients expected the geriatric syndrome to improve. A similar pattern unfolds for the other geriatric syndromes, with outpatients reporting more pessimistic expectations regarding falls (no improvement 75% vs 40.7% for inpatients), pain (no improvement 70.8% vs 18.6% for inpatients), and sleep (no improvement 62.5% vs 29.6% for inpatients, see Fig. 3 and Supplement Table S3). Both in- and outpatients were pessimistic regarding improvements in age-related geriatric syndromes such as loneliness (80.0% outpatients and 53.9% inpatients), cognition (54.5% outpatients and 70.8% inpatients), and incontinence (62.5% outpatients vs 62.9% inpatients); however, due to small sample sizes, these results should be interpreted with caution.

### Geriatric syndromes and quality of life

Finally, we assessed the influence of the number of geriatric syndromes and, in detail, of each geriatric syndrome on physical and mental QoL.

An increasing number of geriatric syndromes was correlated with worse physical (*r* = −0.53, *p* < 0.001) and mental QoL (*r* = −0.43, *p* < 0.001). Notably, this association was even stronger for outpatients (physical *r* = −0.74, *p* < 0.001, mental *r* = −0.64, *p* < 0.001) than for inpatients (physical *r* = −0.45, *p* < 0.001, mental *r* = −0.40, *p* < 0.001). According to Fisher’s Z test, the strength of the correlation is significantly higher in outpatients (*p* < 0.01). We performed regression analyses to understand the link between geriatric syndromes and QoL separately for inpatients and outpatients. For both patient groups, a higher number of geriatric syndromes was associated with lower physical QoL, even when controlling for covariates such as age, gender, GDS, cognition, and Lachs.

For inpatients, a higher number of geriatric syndromes (ß = −5.37, 95% CI [−6.35; −4.40], *p* < 0.001) alone explained 19.5% of the QoL variance; neither gender (ß = 0.37, 95% CI [−3.30; 4.04], *p* = 0.844) nor age (ß = 0.25, 95% CI [−0.05; 0.55], *p* = 0.10) mitigated this effect (ß = −5.42, 95% CI [−6.40; −4.44], *p* < 0.001). With covariates, a higher number of geriatric syndromes remained significantly associated with worse physical QoL (ß = −4.15, 95% CI [−5.20, −3.10], *p* < 0.001) in addition to GDS, MMST and Lachs (Table 2). For outpatients, a similar picture emerged, with a higher number of geriatric syndromes (ß = −8.26, 95% CI [−9.72, −6.81], *p* < 0.001) explaining 49.3% of the variance of lower QoL; this association remained (ß = −8.11, 95% CI [−9.65; −6.57], *p* < 0.001) when adding gender (ß = 0.46, 95% CI [−4.84; 5.75], *p* = 0.864) and age (ß = −0.09, 95% CI [−0.50; 0.32], *p* = 0.667) to the model. With covariates, a higher number of geriatric syndromes remained a significant predictor of lower physical QoL (ß = −5.49, 95% CI [−7.36, −3.63], *p* < 0.001). The same picture was found for mental QoL (Table 2), with a higher number of geriatric syndromes alone explaining 16.7% of QoL variance for inpatients and 42.2% for outpatients. This association remained significant in the second model irrespective of age and gender, and in model 3 after including the other covariates.

Finally, we aimed to assess the influence of individual geriatric syndromes on QoL, repeating the regression analyses separately with each geriatric syndrome and the covariates as predictors for mental or physical QoL. Due to the small sample size of certain geriatric syndromes in outpatients, we performed detailed analyses on the impact of specific geriatric syndromes on QoL primarily for inpatients (Supplement Table S4A), and no results are reported for dysphagia. For inpatients, all geriatric syndromes except cognition were significantly associated with lower physical QoL, with mobility problems (*p* < 0.001, *r* = 0.44), pain (*p* < 0.001, *r* = 0.37), sleep problems (*p* < 0.001, *r* = 0.30), and falls (*p* < 0.001, *r* = 0.29) showing moderate effect sizes (Supplement Table S4). For mental QoL, the associations were weaker, with the highest effect sizes found for loneliness (*p* < 0.001, *r* = 0.37), depressiveness (*p* < 0.001, *r* = 0.35), and cognition (*p* < 0.001, *r* = 0.27). In outpatients, the association between mobility problems (*p* < 0.001, *r* = 0.56), falls (*p* < 0.001, *r* = 0.63), pain (*p* < 0.001, *r* = 0.49), and sleep (*p* < 0.001, *r* = 0.49) as the most prevalent geriatric syndromes was even higher (Supplement Table S4B).

## Discussion

Geriatric syndromes represent disease-overarching syndromes that frequently occur in older age [1]. In the present analysis from the SelfManGer project [25], we aimed to gather insight into geriatric syndromes from the perspective of geriatric patients. For this purpose, we collected data on nine common geriatric syndromes, the amount of limitation patients perceive, the potential they see for improvement, and lastly their association with QoL. For both inpatients and outpatients, we found mobility-related problems to be perceived as most impactful on patients’ daily lives. Even after controlling for sociodemographic information, cognition, mental health, and functional status, a higher number of geriatric syndromes remained linked to worse physical and mental QoL. The presence of each geriatric syndrome negatively impacted QoL, of which mobility problems and pain yielded the strongest impact on physical QoL, while depressiveness, loneliness, and cognitive decline strongly influenced mental QoL. Notably, although they were on average younger and healthier than inpatients, the association between geriatric syndromes and QoL was even stronger in outpatients.

Several previous studies aimed to assess the prevalence of geriatric syndromes; however, due to a lack of consensus on their definition, different studies assessed different geriatric syndromes. For example, Verstraeten et al. [22] looked at polypharmacy and multimorbidity, cognitive deficits, depressive symptoms, nutrition, frailty, and falls. In contrast, Sanford et al. [5] solely researched frailty, weight loss, sarcopenia, and dementia, and Möller et al. [8] further included incontinence, insomnia, and vision impairments. Likewise, many studies combined different sources, combining interviews, standardized questionnaires, and routine data [7]. This heterogeneity in the included geriatric syndromes makes a comparison of studies difficult; however, across all studies, the high prevalence of geriatric syndromes was confirmed: In line with Wang et al. [6] and Bell et al. [7], 90% of our inpatients and 79% of outpatients reported more than one geriatric syndrome. In two cohorts with a comparable age profile to our study, Verstraeten et al. [22] confirmed the high prevalence of geriatric syndromes, stating that most patients had 5 or more geriatric syndromes that rarely occurred in isolation. In our study, nearly half the patients reported four or more geriatric syndromes. Likewise, Alhalaseh et al. [24] found almost all geriatric patients to have geriatric syndromes, ranging from 2.56 geriatric syndromes in younger patients to 3.55 in the oldest patients, matching the responses of our on average younger outpatients and older inpatients. The increasing prevalence of geriatric syndromes with age was confirmed in other studies [2, 5], highlighting the need to understand and address geriatric syndromes in light of the demographic shift.

In addition to determining their presence, the key aim of the present study was to assess the impact geriatric syndromes have on patients. Thus, we asked patients to self-select the geriatric syndromes they experience and also enquired about the restriction and the potential for improvement regarding the geriatric syndromes. To the best of our knowledge, this has not been done before.

From this patient perspective, considerable heterogeneity was present in the interpretation of geriatric syndromes. Some patients reported being strongly impacted by their geriatric syndromes to the point where normal daily activity was no longer possible, whereas other patients were less restricted by the respective geriatric syndrome. This heterogeneity again indicates that a patient-focused approach is needed to individually understand each patient’s experience and expectations. Notably, both in- and outpatients selected mobility-related problems as most frequent and most impactful. This influence of mobility was confirmed in previous studies showing that falls were among the most prevalent geriatric syndromes [7, 14, 22] and were related to increased mortality [14]. Mobility affects well-being and QoL in older adults by enabling independence in daily activities [47]. Additionally, mobility is a pre-requisite for social participation as well as for physical activity [48, 49], all of which are related to physical and mental QoL [50–52], underlining the importance of mobility interventions in clinical care.

While much variability exists in the restriction due to cognitive problems, depressiveness, incontinence, and sleep, pain and falls were further described as relatively restrictive. In inpatients, improvement was expected mostly for pain, while patients were torn regarding mobility-related problems. In outpatients, both mobility problems and pain were assessed more pessimistically. For other geriatric syndromes, patients’ expectations varied. Again, these results indicate that each patient has their own set of geriatric syndromes accompanied by individual expectations and experiences. Notably, both in- and outpatients showed pessimistic expectations regarding cognitive deficits, loneliness, and incontinence, all of which are typically perceived as age-related issues. As expectations regarding aging and health may influence health behavior [53–55], it is crucial to uncover and target these perceptions in clinical care.

In addition to mobility, other frequently reported geriatric syndromes in our study were pain, mentioned by about 60% of patients; sleep problems, mentioned by 42% to 48%; and incontinence, reported by about 35% of inpatients. Little data exist on pain as a geriatric syndrome, with Bell et al. [7] describing a prevalence of 22.3% for moderate to severe pain; notably, their patients were on average younger. While Liu et al. [16] reported a comparable prevalence of 56.4% for sleep problems, Möller et al. [8] presented a slightly higher rate of 60% for insomnia. Regarding urinary incontinence, similar rates between 24 and 40% were previously reported [7, 8, 16, 24]. Notably, Liu et al. [16] showed a significant relation between incontinence and QoL. Depressiveness as a geriatric syndrome was present in 40% of our inpatients but less so in outpatients, which matches the rate of 45% found by Verstraeten et al. [22]. Other studies provided prevalence rates of 60% [14], 30% [7], 28% [24], or 11% when using a questionnaire with a strict cut-off score for severe depression [8]. According to Möller et al. [8], depressive symptoms as geriatric syndromes were linked to increased healthcare utilization.

Cognitive restrictions were present in almost a third of both in- and outpatients, and again a third of outpatients perceived these as the most impactful geriatric syndromes. Wang et al. [13] linked cognitive deficits with hospitalization and nursing home admission, and Gómez-Ramos et al. [14] even proposed a link to increased mortality. The presence of cognitive deficits is often based upon cognitive tests [8, 22], leading to higher prevalence rates compared to our study, although Bell et al. [7] reported similar rates of 25.5%. The differences in the prevalence of cognitive deficits may be based on different tests and cut-off scores. Of note, due to the nature of the overall study, persons with severe cognitive deficits and insomnia were excluded from participation.

Overall, geriatric syndromes are an important cornerstone in geriatric care not only due to their increasing prevalence in advancing age, but also due to their relation with health outcomes and QoL [13, 14, 16, 56]. Using regression analysis, we showed a higher number of geriatric syndromes to be associated with worse physical and mental QoL even when controlling for age, gender, cognition, mood, and functional health. In a similar fashion, Liu et al. [17] found geriatric syndromes to be linked with QoL even when considering concrete illnesses. Likewise, in their review, Wang et al. [13] link geriatric syndromes with hospital admission even when controlling for specific diseases. These results suggest that geriatric syndromes contribute to well-being above and beyond simple health status and carry information that is not covered in singular illnesses or specific assessments. The impact of geriatric syndromes further becomes evident in the comparably high variance explanation, ranging from 15 to 20% for mental and physical QoL in inpatients and even higher in outpatients. These results are comparable to previous research in older diabetic patients, where geriatric syndromes alone explained 25% of physical QoL in comparison to only 4% explained by explicit comorbid diseases. A similar picture emerged for mental QoL [16]. These results again highlight the high informational value of geriatric syndromes and spotlight them as targets for clinical interventions to improve QoL.

In addition to a higher number of geriatric syndromes contributing to lower QoL, we looked at the influence of individual geriatric syndromes. Similar to Liu et al. [16], our patients reported lower physical and mental QoL for all geriatric syndromes in comparison to those patients without the respective geriatric syndromes. For physical QoL, effect sizes were moderate for mobility-related problems, pain, and sleep, whereas mental QoL was most closely related to depressiveness, cognition, and loneliness. In previous studies, individual geriatric syndromes such as depressiveness, cognition, and insomnia were linked to lower QoL [11, 16, 56]. These results indicate a relation between the presence of overarching geriatric syndromes and worse well-being in older patients.

Psychological theories may aid in the interpretation of these results. Notably, in contrast to concrete illnesses which may be attributed to a specific cause and interpreted as out of control, overarching geriatric syndromes cannot be assigned to a singular cause, making them more diffuse in their control perception and attribution [57]. Likewise, their global impact on various areas of life may further reduce functionality and thus affect QoL more broadly than singular illnesses with a restricted set of symptoms. This approach of attribution and aging expectations should be explored in depth in future studies to understand the mechanisms with which geriatric syndromes impact health and well-being of geriatric patients. Notably, despite lower prevalence rates, the association between geriatric syndromes and QoL was even stronger in objectively younger and healthier outpatients. Both the Social Cognitive Theory [58] and the Social Comparison Theory [59] propose an influence of the surrounding social network on well-being and attitudes, such as the interpretation of health and age-related changes. A comparison with the health and ability of peers may be influential in the sense that at a certain age, most peers also suffer from age-related decline and illness, thus normalizing this decline. In contrast, at a younger age, peers may not yet show a decline in functional ability and health, leading to a more detrimental interpretation of one’s own worsening health [60]. Likewise, the Stereotype Embodiment Theory proposes aging as a partially social construct, dictating that interpretations of the aging process are rooted in age-related stereotypes that form across the lifespan and become activated when confronted with the own aging process [61]. In accordance, coping theory such as the SOC model of selective optimization with compensation [62] proposes that an adjustment of goals and behavior is necessary proportional to the current abilities; the strong impact of geriatric syndromes in outpatients may be rooted in the shorter time frame these younger patients had to cope with health changes and optimize their behavioral approaches. These perceptions of aging may change over time, shifting towards a perspective of physical and social losses with advancing age [63]. This is also evident in the fact that while our outpatients showed better physical health and fewer medications and diagnoses than our inpatients, their GDS and mental QoL are comparable. These findings suggest that the presence of geriatric syndromes may be related to a negative mood and interpretation of younger patients’ health status. Likewise, resilience may play a role in the influence of geriatric syndromes on QoL. Resilience describes the ability of a person or an organism to recover from stressful events or decline [64]. In previous studies, resilience was lower in middle-aged adults than in older adults [65], while higher resilience positively influenced both the perception of health [66] and QoL [65]. Thus, resilience may serve as a buffer indicating that our older inpatients believe themselves fit to handle health circumstances. These findings highlight the need to incorporate attitudes towards aging in clinical care, as these may shape well-being and behavior in negative ways, but also provide potential for growth and protection [63, 67, 68]. Future studies should incorporate the influence of aging cognitions, coping strategies, resilience, and age stereotypes to elucidate their effect on QoL.

Several implications for clinical application can be drawn from the present data. First, in concordance with previous results [13, 17], our data showed that patients’ perspectives on geriatric syndromes convey health-related information that influenced important clinical outcomes such as QoL above and beyond other health measures. In clinical care, the assessment of geriatric syndromes is oftentimes based on objective measures and medical expertise, yet the present data highlight the importance of incorporating the patient perspective. This patient perspective is crucial, especially as individual expectations and attributions dictate behavior.

Especially in light of the demographic shift that brings with it constraints in the care for older persons presenting with multimorbidity, an efficient approach is essential. Therefore, understanding the syndromes patients themselves perceive as most impactful helps guide clinical care in a targeted manner. Additionally, understanding psychosocial mechanisms that influence the perception of geriatric syndromes and age-related health changes provides anchor points for counseling and psychological support that may aid patients in navigating health changes in advancing age.

Overall, the main strength of this study is its patient-centered approach on prevalence and perception of geriatric syndromes in an oftentimes under-researched patient population. It further refines results on the influence of geriatric syndromes on QoL and thus enriches previous research by providing a patient perspective on a highly prevalent but not yet understood cornerstone of geriatric healthcare. Still, the study is not free of limitations.

Its cross-sectional design does not allow for an interpretation of causality. Although the association between geriatric syndromes and QoL was confirmed in previous studies, longitudinal data are required to fully understand their link. Likewise, the mechanisms by which geriatric syndromes influence QoL on top of objective health cannot be uncovered from the present data; instead, information on aging attitudes, coping, and attribution should be included in future studies to provide an encompassing understanding of geriatric syndromes and their relation with psycho-social and health-related variables. Additionally, the direction of effects should be considered in future studies: while the impact of geriatric syndromes on QoL becomes clear in this and previous research, the reverse direction should also be considered. Low QoL, especially in the mental domain, may momentarily heighten the experience of geriatric syndromes such as pain or sleep problems, as well as dictate the expectations regarding these syndromes [66]. While the patient perspective aims to specifically uncover the subjective experience of each patient, future studies should consider bidirectional effects in longitudinal analyses.

Additionally, the data were collected in German clinical care, thus its generalizability to other regions and nations is limited. Cultural expectations as well as healthcare standards may influence the perception of geriatric syndromes for patients across the globe. As the data are based on patients’ experiences, some sample sizes—for example, for dysphagia—are small, hindering robust analyses. Thus, some results should be interpreted with caution. Instead, this study aims to guide future research in the area of patient-centered approaches on geriatric syndromes and their impact by providing first insights into an under-explored topic. Additionally, patients with dementia, acute delirium, or severe depression were excluded from participation in this study, potentially introducing a bias towards geriatric patients with better functionality. With regard to the influence of geriatric syndromes above and beyond health, it is crucial to understand how they impact patients with dementia, and future studies should target the assessment of geriatric syndromes in patients with cognitive deficits. This is especially important as research links cognitive geriatric syndromes with increased mortality risk [15].

Likewise, across studies, a different interpretation of geriatric syndromes exists due to a lack of consensus on which geriatric syndromes should be included. Therefore, when interpreting the results and comparing them with other research, a potential variation in included geriatric syndromes must be kept in mind. Certain differences in the prevalence and impact of geriatric syndromes may stem from differing types of included geriatric syndromes.

Lastly, although the primary goal of our study was to provide a patient perspective on geriatric syndromes, self-reported information always carries a risk of bias. The presented geriatric syndromes are not based on medical expertise but rather on patients’ own perception of their health. While the patient perspective is essential in clinical research [27, 69, 70], the results may differ from previous studies where geriatric syndromes were assessed with standardized measures. Future studies should compare expert- and patient-given assessments to understand how they relate to each other and to QoL.

## Conclusion

Geriatric syndromes are disease-unspecific, overarching symptom clusters that may be useful in the treatment of older adults, where multimorbidity renders a disease-focused approach ineffective. From a patient’s perspective, geriatric syndromes are common, with mobility-related problems, sleep disturbances, pain, and cognitive impairment rated as particularly prevalent. Although much variability exists regarding the amount of restriction patients experienced and the potential they see for improvement, a higher number of geriatric syndromes was associated with worse physical and mental QoL even when controlling for sociodemographic and health-related information. In future studies, longitudinal data may provide an in-depth understanding of causal relationships between geriatric syndromes and QoL. The addition of further covariates such as aging perceptions and motivations may help uncover the underlying mechanisms of this association. A deeper understanding of the most impactful geriatric syndromes and their associations with well-being can guide geriatric care in a more effective and patient-centered manner.

## Supplementary Information

Below is the link to the electronic supplementary material.

Supplementary file1 (PDF 757 KB)

## Data Availability

All study materials are available from Schönenberg, A., Heimrich, K. G., Prell, T., et al. (2025). Data on Self-Management of Geriatric Syndromes. osf.io/a68x5 [28]. Of note, due to the potential sensitive nature of the data, the datasets are only available after request.
