# Supplementary material for: Patient-reported geriatric syndromes and their association with quality of life: findings from a cross-sectional study in German older adults
Source: Eur Geriatr Med. 2025 Oct 16;17(2):721–33. doi: 10.1007/s41999-025-01332-7 (PMC13109236; doi:10.1007/s41999-025-01332-7)
Supplement: Supplementary file 1 — Supplementary file1 (PDF 757 KB) [file 41999_2025_1332_MOESM1_ESM.pdf]

Supplementary Materials

Supplement Table 1. Presence of and restriction due to geriatric syndromes

Supplement Table 2. Syndrome presence based on gender

Supplement Table 3. Expected improvement of geriatric syndromes

Supplement Table 4. Group comparison for Quality of Life depending on Syndrome Presence

Supplement Table 1. Presence of and restriction due to geriatric syndromes

Syndrome	Present in N (%)	Most restrictive* N (%)	Restriction M (SD)	Syndrome	Present in N (%)	Most restrictive* N (%)	Restriction M (SD)
Mobility				Pain			
Inpat	396 (83.9)	211 (53.3)	65.6 (27.5)	Inpat	282 (59.6)	71 (25.2)	60.3 (24.7)
Outpat	95 (68.8)	60 (63.2)	50.7 (26.2)	Outpat	78 (56.5)	24 (30.8)	43.7 (30.2)
Falls				Incont.			
Inpat	337 (71.4)	83 (24.6)	59.0 (24.1)	Inpat	167 (35.4)	35 (30.0)	52.0 (29.3)
Outpat	58 (42.0)	8 (13.8)	45.0 (26.6)	Outpat	33 (23.9)	8 (24.2)	18.5 (12.6)
Cognition				Sleep			
Inpat	132 (28.0)	13 (9.9)	37.3 (33.5)	Inpat	202 (42.8)	28 (13.9)	52.3 (28.9)
Outpat	40 (29.0)	13 (32.5)	24.9 (28.3)	Outpat	67 (48.6)	16 (23.9)	26.2 (22.6)
Depress.				Dysphag			
Inpat	187 (39.6)	11 (5.9)	48.6 (34.6)	Inpat	42 (8.9)	4 (9.5)	47 (38.2)
Outpat	21 (15.2)	3 (14.3)	35.0 (26.0)	Outpat	10 (7.25)	1 (10.0%)	26.2 (22.5)
Loneliness				N=511 vs 155, *N= 472 vs 145			
Inpat	100 (21.2)	13 (13.0)	42.1 (22.9)				
Outpat	22 (15.9)	5 (22.7)	62.0 (26.8)				

Most restrictive = “Which of the presented syndromes is most restrictive to you in your daily life?” Restriction = on a scale of 0 = no restriction to 100 = fully restricted, how restricted are you due to this syndrome?

Supplement Table 2. Syndrome presence based on gender

		Entire Cohort		Inpatients (N = 511)	
Syndrome		Count (% all patients, % by gender)	Chi²	Count (% all patients, % by gender)	Chi²
Mobility	Female	320 (65.2, 74.8)	.467	262 (66.2, 78.2)	.673
	Male	171 (34.8, 71.9)		134 (33.8, 76.1)	
Falls	Female	264 (66.8, 61.7)	.112	224 (66.5, 66.9)	.614
	Male	131 (33.2, 55.0)		113 (33.5, 65.2)	
Cognition	Female	103 (59.9, 24.1)	.194	80 (60.6, 23.9)	.200
	Male	69 (40.1, 29.0)		52 (39.4, 29.6)	
Depress	Female	132 (64.7, 30.8)	.750	121 (64.7, 36.1)	.833
	Male	72 (25.3, 30.3)		66 (35.3, 37.5)	
Lonely	Female	84 (68.9, 19.6)	.287	70 (70.0, 20.9)	.355
	Male	38 (31.2, 16.0)		30 (30.0, 17.1)	
**Pain**	**Female**	**248 (68.9, 57.9)**	**.009**	**196 (69.5, 58.5)**	**.047**
	**Male**	**112 (31.1, 47.1)**		**86 (30.5, 48.9)**	
Incontinence	Female	135 (67.5, 31.5)	.292	113 (67.7, 33.7)	.549
	Male	65 (32.2, 27.3)		54 (32.3, 30.7)	
Sleep	Female	181 (57.3, 42.3)	.209	135 (66.8, 40.3)	.693
	Male	88 (32.7, 37.0)		67 (33.2, 38.1)	
**Dysphag**	**Female**	**25 (48.1, 5.8)**	**.017**	22 (52.4, 6.6)	.088
	**Male**	**27 (51.9, 11.3)**		20 (47.6, 11.4)	
		**Mean (SD)**	***p, r***	**Mean (SD)**	***p, r***
SyndromNum	Female	3.50 (1.83)	.062,	3.65 (1.78)	.341
	Male	3.25 (1.86)	.072	3.53 (1.82)	.042
SRH	Female	**2.84 (1.15)**	**.016,.111**	**2.84 (1.15)**	**.016**
	Male	**2.58 (1.18)**		**2.58 (1.18)**	**.111**
SRH = Self-Rated Health, SyndromNum = Number of geriatric syndromes, Dysphag = Dysphagia, Depress = Depressive Symptoms					

Supplement Table 3. Expected improvement of geriatric syndromes

Syndrome	Improvement Expected	Improvement Not expected	Undecided
Mobility	95 (35.6%)	132 (49.4%)	40 (15.0%)
Inpatient	86 (41.6%)	83 (40.1%)	38 (18.3%)
Outpatient	9 (15.0%)	49 (81.7%)	2 (3.3%)
Falls	36 (40.5%)	39 (43.8%)	14 (15.7%)
Inpatient	34 (42.0%)	33 (40.7%)	14 (17.3%)
Outpatient	2 (25.0%)	6 (75.0%)	0
Cognition	1 (4.2%)	17 (70.8%)	6 (25.0%)
Inpatient	1 (9.1%)	6 (54.5%)	4 (36.4%)
Outpatient	0 (0)	11 (84.6%)	2 (15.4%)
Depressiveness	6 (50.0%)	6 (50.0%)	0
Inpatient	5 (55.6%)	4 (44.4%)	0
Outpatient	1 (33.3%)	2 (66.7%)	0
Loneliness	4 (22.2%)	11 (61.1%)	3 (16.7%)
Inpatient	3 (28.1%)	7 (53.9%)	3 (18.0%)
Outpatient	1 (20.0%)	4 (80.0%)	0
Pain	45 (47.9%)	30 (31.9%)	19 (20.2%)
Inpatient	39 (55.7%)	13 (18.6%)	18 (25.7)
Outpatient	6 (25.0%)	17 (70.8%)	1 (4.2%)
Incontinence	11 (25.6%)	27 (62.8%)	5 (11.6%)
Inpatient	8 (22.9%)	22 (62.9%)	5 (14.2%)
Outpatient	3 (37.5%)	5 (62.5%)	0
Sleep	16 (37.2%)	18 (41.9%)	9 (20.9%)
Inpatient	12 (44.4%)	8 (29.6%)	7 (26.0%)
Outpatient	4 (25.0%)	10 (62.5%)	2 (12.5%)
Dysphagia	2 (40.0%)	1 (20.0%)	2 (40.0%)
Inpatient	2 (50.0%)	1 (25.0%)	1 (25.0%)
Outpatient	0	0	0
Expected = score > 50, not expected = score < 50, undecided = 50			

Supplement Table 4. Group comparison for Quality of Life depending on Syndrome Presence

Syndrome		Phys M (SD)	p	EffSize	Ment M (SD)	p	EffSize
**A** **Inpatients -** Quality of life depending on syndrome presence (group comparison), u test							
Mobility	No	65.6 (18.9)	< .001	0.44	72.8 (14.4)	.006	0.18
	Yes	49.6 (20.9)			67.0 (17.2)		
Falls	No	60.3 (21.0)	< .001	0.29	72.1 (18.0)	.001	0.22
	Yes	49.4 (20.9)			66.3 (15.7)		
Cognition	No	53.9 (21.3)	0.146	0.09	70.5 (15.7)	< .001	0.27
	Yes	50.7 (22.1)			61.8 (17.9)		
Depress	No	55.5 (20.0)	.001	0.20	72.0 (15.4)	< .001	0.35
	Yes	48.8 (23.4)			61.5 (16.9)		
Lonely	No	54.5 (21.5)	.003	0.20	70.2 (16.5)	< .001	0.37
	Yes	46.9 (20.5)			60.2 (15.4)		
Pain	No	60.8 (22.5)	< .001	0.37	70.4 (17.7)	.005	0.15
	Yes	47.0 (18.6)			66.6 (15.8)		
Incontinence	No	54.6 (20.5)	.007	0.15	70.1 (16.1)	.002	0.18
	Yes	50.0 (23.2)			64.6 (17.3)		
Sleep	No	57.6 (21.4)	< .001	0.30	70.4 (15.8)	.001	0.17
	Yes	46.2 (19.9)			65.1 (17.6)		
**B** **Outpatients -** Quality of life depending on syndrome presence (group comparison), u test							
**Syndrome**		**Phys M (SD)**	**p**	**EffSize**	**Ment M (SD)**	**p**	**EffSize**
Mobility	No	72.4 (14.7)	< .001	0.56	74.4 (11.8)	< .001	0.42
	Yes	53.0 (19.8)			62.3 (18.1)		
Falls	No	68.8 (17.7)	< .001	0.63	70.9 (14.7)	< .001	0.35
	Yes	47.4 (17.1)			60.8 (18.5)		
Cognition*	No	63.5 (19.1)	0.081	0.21	70.6 (14.3)	< .001	0.41
	Yes	55.3 (22.4)			56.3 (19.6)		
Depress*	No	63.3 (19.9)	.009	0.39	69.3 (16.1)	< .001	0.55
	Yes	49.8 (18.2)			53.2 (15.4)		
Lonely*	No	63.5 (19.3)	.003	0.47	70.1 (15.1)	< .001	0.64
	Yes	46.2 (20.8)			49.4 (16.4)		
Pain	No	69.3 (17.4)	< .001	0.49	72.5 (15.6)	< .001	0.38
	Yes	2.8 (19.6)			61.8 (16.5)		
Incontinence*	No	63.1 (19.8)	.069	0.23	68.9 (16.2)	.012	0.31
	Yes	55.7 (20.9)			59.0 (18.1)		
Sleep	No	68.5 (17.2)	< .001	0.49	73.2(13.0)	< .001	0.48
	Yes	51.7 (20.1)			59.0 (18.1)		

Ment = mental subscale of WHOQOL-Bref quality of life questionnaire
Phys = physical subscale of WHOQOL-Bref quality of life questionnaire
Effsize: rank biserial correlation
*Note: Due to small sample sizes, grey cells in table 4b should be interpreted with caution.
